# The Impact of Pathologic Upgrading of Gleason Score 7 Prostate Cancer on the Risk of the Biochemical Recurrence after Radical Prostatectomy

**DOI:** 10.1155/2018/4510149

**Published:** 2018-04-30

**Authors:** Juhyun Park, Sangjun Yoo, Min Chul Cho, Min Hyun Cho, Chang Wook Jeong, Ja Hyeon Ku, Cheol Kwak, Hyeon Hoe Kim, Hyeon Jeong

**Affiliations:** ^1^Department of Urology, SMG-SNU Boramae Medical Center, Seoul National University, College of Medicine, Seoul, Republic of Korea; ^2^Department of Urology, Seoul National University Hospital, Seoul National University, College of Medicine, Seoul, Republic of Korea

## Abstract

**Objective:**

To investigate the impact of pathologic upgrading of Gleason score (GS) 7 prostate cancer on the risk of the biochemical recurrence.

**Materials and Methods:**

A total of 1678 patients with postoperative GS 7 prostate cancer without lymph node metastasis were reviewed retrospectively. The patients were categorized into four groups depending on pathologic upgrading: upgraded GS 3+4, nonupgraded GS 3+4, upgraded GS 4+3, and nonupgraded GS 4+3. Kaplan-Meier multivariate model was created.

**Results:**

The mean age was significantly higher in the nonupgraded GS 4+3 group than in other groups, whereas the mean prostate-specific antigen (PSA) level was lower in the upgraded GS 3+4 group. Pathologic findings, such as extracapsular extension, seminal vesical invasion, and the surgical margin rate, were different from each other. Five-year biochemical recurrence-free survival rate was 85%, 73%, 69%, and 60% in upgraded GS 3+4, nonupgraded GS 3+4, upgraded GS 4+3, and nonupgraded GS 4+3 group, respectively. There was significant difference between the nonupgraded 4+3 and upgraded 4+3 group, as well as between upgraded 3+4 and nonupgraded 3+4 group. However, the two middle patient groups, that is, the nonupgraded GS 3+4 group and the upgraded GS 4+3 group, did not show the statistical difference (Log-rank test, *p* value = 0.259).

**Conclusion:**

The information on pathologic upgrading in the biopsy reports of patients could help to provide more detailed analysis for the biochemical recurrence of GS 7 prostate cancer.

## 1. Introduction

Ever since Gleason was able to predict mortality rates based on prostate cancer histology, followed by the establishment of the Gleason scoring system in the mid-1960s, this grading system has become an important prognostic determinant of prostate cancer [1]. Despite modifications to and revision of this system, the ability to accurately determine the Gleason score (GS) and prostate cancer grading is critical in forecasting patient prognosis and in determining the treatment policy to be adopted [2, 3]. Nevertheless, the traditional prostate cancer grading system is known to have several limitations, one of which is the heterogeneity of pathologic GS 7 prostate cancer. It has been indicated in previous studies that numerous outcomes are associated with GS 3+4 and GS 4+3 prostate cancer, despite both constituting GS 7 prostate cancer [4–7].

Recently, a new grading system was introduced and was demonstrated to be useful following multicentered, multinational validation. The new grading system involves a five-point-scale grade group system, according to which GS 3+4 and GS 4+3 are classified as grade groups 2 and 3, respectively. This prevents confusion arising from the misconception that GS 3+4 and GS 4+3 prostate cancer both eventually become GS 7 prostate cancer and makes it easier to forecast patient prognosis and the need for additional treatment after surgery [3, 8, 9]. However, the equal division of GS 7 prostate cancer between GS 3+4 and GS 4+3 prostate cancer is not feasible. The category is perceived to be continuous and should be sorted according to increments in the percentage of GS 4 prostate cancer instead [10, 11].

Sauter et al. suggested a new methodology with which to analyze GS 7 prostate cancer using the quantitative GS 4 histology proportion [10]. However, it is difficult to adopt a quantitative Gleason grading system, because it is almost impossible to review all radical prostatectomy specimens using quantitative analysis, whereas Corcoran et al. reported that different prognoses were attributed to the same cases of GS 4+3 cancer as per the pathologic upgrading [12]. Pathologic upgrading from GS 3+4 to GS 4+3 prostate cancer can be assumed to be a discrepancy between the biopsy and final pathology findings according to the degree of GS 4 containment [6, 10, 13].

In this study, an alternative classification method was proposed whereby GS 7 prostate cancer was divided into four categories based on the pathologic upgrading information. And the impact of pathologic upgrading of Gleason score (GS) 7 prostate cancer on the risk of the postoperative biochemical recurrence was evaluated.

## 2. Methods

Between April 1996 and November 2016, 2984 consecutively presenting patients underwent a prostate biopsy and radical prostatectomy at two medical centers in Korea. Of these, 1678 (56%) GS 7 prostate cancer patients without lymph node metastasis were included in the current study. A review was conducted of retrospectively obtained clinicopathological data taken from the medical records. Radical prostatectomy was performed by several surgeons during the study period. The biopsy and prostatectomy specimens were assessed by experienced genitourinary pathologists.

The Institutional Review Board approved this study protocol. The study conformed to the tenets of the Declaration of Helsinki. As the present study was carried out retrospectively, the need to obtain written informed consent from the patients was waived by the Institutional Review Board. Personal identifiers of patients were removed completely and the data were analyzed anonymously.

### 2.1. Study Variables

The following variables were included in current analysis: age, body mass index (BMI), preoperative prostate-specific antigen (PSA) test level result, GS, clinical stage, extracapsular extension, seminal vesicle invasion, surgical margin status, and biochemical recurrence rate. The last one was defined as a PSA value ≥ 0.2 ng/mL after two consecutive measurements had been taken [14]. In general, PSA was checked postoperatively at three, six, and 12 months and thereafter annually in the absence of biochemical recurrence.

The preoperative PSA level was categorized as <10 ng/dL, 10–20 ng/dL, and >20 ng/dL, and the clinical stages were classified as T1c/T2a, T2b/c, and T3-4. Patients were divided into four groups according to pathologic upgrading, namely, upgraded GS 3+4, nonupgraded GS 3+4, upgraded GS 4+3, and nonupgraded GS 4+3. Kaplan-Meier and multivariate analysis were undertaken to examine the influence of pathologic upgrading of GS 7 prostate cancer on the risk of biochemical recurrence.

### 2.2. Statistical Analysis

The variables are presented as mean ± standard deviation. Statistically significant differences regarding the preoperative parameters in the subgroups were analyzed using analysis of variance and chi-square and independent* t*-tests. To evaluate predictors of pathologic upgrading status and biochemical recurrence, logistic regression analysis was performed. The* p*-values were two-sided. A* p* value of <0.050 was considered to be statistically significant. Statistical analysis was performed using SPSS® version 22.0 (IBM, Armonk, USA).

## 3. Results

Mean age, preoperative PSA level, clinical stage, pathologic features, and the biochemical recurrence rate in the two GS7 prostate cancer groups (GS 3+4 and GS 4+3) were found to differ significantly, with the exception of the positive surgical margin rate (Table 1).

Statistically significant differences in the clinicopathological parameters were also evident using this four-group categorization of pathologic GS 7 prostate cancer. The mean age of the patients was significantly higher in the nonupgraded GS 4+3 group than other groups, whereas the mean PSA level was lower in the upgraded GS 3+4 group than other groups. The PSA level and clinical stage differed significantly in each group. Pathologic findings, such as extracapsular extension, seminal vesical invasion, and the surgical margin rate were different from each other in each group. Biochemical recurrence-free survival was found to be the most favorable in the upgraded GS 3+4 group and the least so in the nonupgraded GS 4+3 group (Table 2).

**Figure 1:**
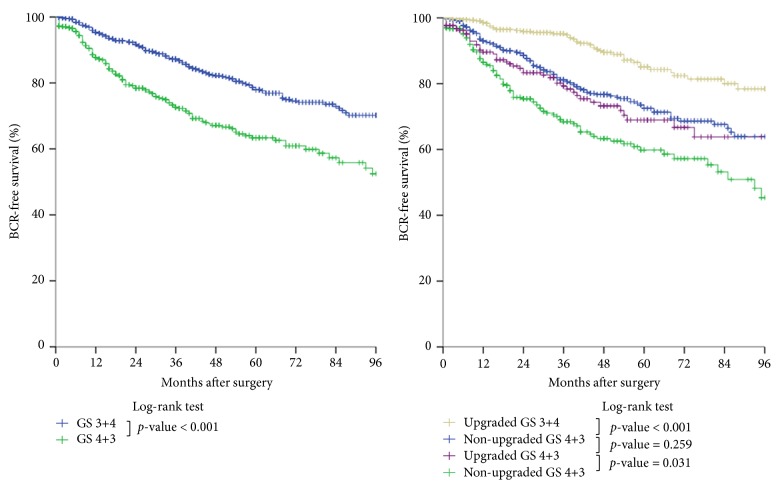
Biochemical recurrence-free survival of pathologic GS 7 prostate cancer patients according to classical two-group categorization and novel four-group categorization. BCR: biochemical recurrence; GS: Gleason score.

Five-year biochemical recurrence-free survival rate was 85%, 73%, 69%, and 60% in upgraded GS 3+4, nonupgraded GS 3+4, upgraded GS 4+3, and nonupgraded GS 4+3 group, respectively. Greater biochemical recurrence-free survival was demonstrated in the upgraded GS 3+4 group compared to that in the original GS 3+4 group (78%), whereas lower biochemical recurrence-free survival was found in the nonupgraded GS 4+3 group, compared to the original GS 4+3 group (63%) (Figure 1).

A significant hazard ratio (HR) was reported in relation to a PSA of >20 ng/mL and the surgical margin status in multivariate analysis. The most optimum prognosis for biochemical recurrence-free survival was assigned to the upgraded GS 3+4 group and the least favorable prognosis was ascribed to the nonupgraded GS 4+3 group. Using nonupgraded GS 3+4 group as a reference for the multivariate models, there was significant difference between the nonupgraded 4+3 and upgraded 4+3 group, as well as between upgraded 3+4 and nonupgraded 3+4 group. However, the two middle patient groups, that is, the nonupgraded GS 3+4 group and the upgraded GS 4+3 group, did not show the statistical difference (HR 1.204,* p* value = 0.274) (Figure 1, Table 3).

## 4. Discussion

An alternative classification method was applied in the current study whereby GS 7 prostate cancer was classified according to four-group categories based on the information on pathologic upgrading. The influence of the pathologic upgrading of GS 7 prostate cancer on the risk of biochemical recurrence was then examined.

Provided that sufficient biopsy information was available, this classification system was relatively simple and easy to use. The most favorable prognosis for biochemical recurrence-free survival was found in the upgraded GS 3+4 group and the least favorable prognosis was reported in the nonupgraded GS 4+3 group and intermediate nonupgraded GS 3+4 and upgraded GS 4+3 groups. The risk of biochemical recurrence was 0.5 times higher in the upgraded GS 3+4 group than it was in the nonupgraded GS 3+4 group and 1.6 times higher in the nonupgraded GS 4+3 group than it was in the nonupgraded GS 3+4 group (Table 3).

Consequently, this novel way of classifying GS 7 prostate cancer can be used to prevent unnecessary examination in the follow-up period in the group with the finest prognosis and prepare appropriate management strategy in the group with the poorest prognosis for biochemical recurrence-free survival [15].

It was interesting that the expected clinicopathological features in each group according to pathologic upgrading were actually observed in the four groups. Because the pathologic upgrading could occur according to a portion of the GS 4 prostate histology in final pathology, the priority determination of the four groups was relatively easy in aspect of prognosis. In other words, a low GS 4 prostate histology was indicative of ≤GS 3+4 prostate cancer. Conversely, a higher GS 4 prostate histology was indicative of ≥ GS 4+3 prostate cancer [10, 13].

There was no significant difference in the postoperative biochemical recurrence-free survival between the intermediate nonupgraded GS 3+4 group and upgraded GS 4+3 groups (Figure 1). This could be explained by the hypothesis that determining the accurate proportion of GS 3 and GS 4 prostate histology in GS 7 prostate cancer was not so easy a task as might be supposed. When the GS 4 glands were very small or comprised almost the entire pathology, it was relatively easy to determine the final pathologic GS, whereas it was very difficult to do so when the GS 4 glands were widely distributed, that is, by 50%. In addition, the distributional pattern of the GS 3 and 4 glands was even more complicated in the case of prostate cancer with multiple tumor nodules. The tumor multiplicity of radical prostatectomy specimen is a common pathologic feature [16]. Consequently, the intermediate two groups, nonupgraded GS 3+4 group and upgraded GS 4+3 groups, could not help avoiding the confused categorization since they were classically divided into GS 3+4 group and GS 4+3 group.

A PSA of >20 ng/mL or a surgical margin status was defined on multivariate analysis as significant prognostic factors for biochemical recurrence-free survival, while the rest of the parameters were not (Table 3). It was assumed in the current study that only lymph node-negative GS 7 prostate cancer was included. Although extracapsular extension and seminal vesicle invasion were not observed to be significant predictors of biochemical recurrence in current study, such findings were considered as major prognostic parameters that have been constantly identified in previous reports [17–19].

The new Gleason grade grouping system was proposed recently and has been validated by several major centers treating numerous prostate cancer patients who have undergone various treatment modalities, such as radical prostatectomy, radiation, and androgen deprivation therapy. The use of the new Gleason grade grouping system is already recommended in several guidelines [3, 9, 20–22].

As mentioned previously, according to the new classification system, GS 3+4 and GS 4+3 prostate cancer are now categorized as grade groups 2 and 3 prostate cancer [3]. Paradoxically, this new grade group classification just divided GS 7 prostate cancer as two typical groups, but could not suggest more detailed prognostic group categorization. The objective of identifying and classifying the various characteristics of prostate cancer pathology is to be able to better predict patient prognosis and to evaluate the need for further treatment [15]. Thus, the novel four-group classification of GS 7 prostate cancer which was proposed in the current study was easy to use and more suitable for clinical utilization than traditional classification when the biopsy information was available.

There were several limitations to the current study. The primary one was that it involved retrospective analysis. It was also a just two-institute study that targeted the Korean population, so validation analysis would be required for multicentered and multinational studies. The tertiary GS 5 histology from the biopsy and final pathologic specimen were not used in the four-group categorization because adding tertiary GS 5 histology would increase the complexity of categorization model and make it difficult to use in a real clinical situation [23, 24]. Nevertheless, this study was worthwhile as proposed; this novel four-group GS 7 prostate cancer classification can be used to overcome the grade group system limitation of being unable to categorize prognosis groups to a greater degree and in greater detail [3].

## 5. Conclusion

The information on pathologic upgrading in the biopsy reports of patients could help to provide more detailed analysis for the biochemical recurrence of GS 7 prostate cancer. Future studies are required to properly categorize the middle two mixed prostate cancer groups in aspect of the prognosis after surgery.

## Figures and Tables

**Table 1:** Patients' characteristics according to the classic categorization of pathologic Gleason score 7 prostate cancer.

	GS 3+4	GS 4+3	*p* value
*N*	1094	584	
Mean age	66.6 ± 6.5	67.4 ± 6.4	0.010
Preoperative PSA	11.3 ± 11.2	15.4 ± 21.1	<0.001
Preoperative PSA level			
<10	691 (69.9%)	303 (51.9%)	<0.001
10–20	286 (26.1%)	155 (26.5%)	
>20	117 (10.7%)	126 (21.6%)	
Clinical stage			
T1c/T2a	971 (88.8%)	486 (83.2%)	0.001
T2b/c	99 (9.0%)	68 (11.6%)	
T3-4	24 (2.2%)	30 (5.1%)	
Pathologic finding			
ECE+	365 (33.4%)	278 (47.6%)	<0.001
SVI+	87 (8.0%)	90 (15.4%)	<0.001
Margin+	416 (38.0%)	238 (40.8%)	0.149
Biochemical recurrence	170 (15.5%)	154 (26.4%)	<0.001

ECE: extracapsular extension; GS: Gleason score; PSA: prostate-specific antigen; SVI: seminal vesicle invasion.

**Table 2:** The four-group categorization of pathologic Gleason score 7 prostate cancer according to Gleason score upgrading after a radical prostatectomy.

	Upgraded GS 3+4	Nonupgraded GS 3+4	Upgraded GS 4+3	Nonupgraded GS 4+3	*p* value
*N*	458	636	216	368	
Mean age	66.7 ± 6.4	66.5 ± 6.6	66.5 ± 6.5	68.0 ± 6.2^*∗*^	0.003
Preoperative PSA	9.4 ± 7.6^*∗*^	12.6 ± 13.0	14.5 ± 26.4	15.9 ± 17.5	<0.001
Preoperative PSA level					
<10	320 (69.9%)	371 (58.3%)	124 (57.4%)	179 (48.6%)	<0.001
10–20	108 (23.6%)	178 (28.0%)	53 (24.5%)	102 (27.7%)	
>20	30 (6.6%)	87 (13.7%)	39 (18.1%)	87 (23.6%)	
Clinical stage					
T1c/T2a	411 (89.7%)	560 (88.1%)	191 (88.4%)	295 (80.2%)	0.001
T2b/c	36 (7.9%)	63 (9.9%)	18 (8.3%)	50 (13.6%)	
T3-4	11 (2.4%)	13 (2.0%)	7 (3.2%)	23 (6.3%)	
Pathologic finding					
ECE+	134 (29.9%)	231 (36.3%)	94 (43.5%)	184 (50.0%)	<0.001
SVI+	14 (3.1%)	73 (11.5%)	27 (12.5%)	63 (17.1%)	<0.001
Surgical margin+	152 (33.2%)	264 (41.5%)	86 (39.8%)	152 (41.3%)	0.027
Biochemical recurrence	46 (10.0%)	124 (19.5%)	49 (22.7%)	105 (28.5%)	<0.001

*∗* denotes statistical significance in comparison with the other three groups; ECE: extracapsular extension; GS: Gleason score; PSA: prostate-specific antigen; SVI: seminal vesicle invasion.

**Table 3:** Multivariate analysis of biochemical recurrence-free survival based on the four-group categorization of pathologic Gleason score 7 prostate cancer.

Variables	Biochemical recurrence-free survival	
	HR (95% CI)	*p* value
Age	1.01 (0.99–1.03)	0.349
PSA range		
<10	Reference	
10–20	1.19 (0.911–1.54)	0.206
20	1.50 (1.11–2.03)	0.008
Pathologic findings		
ECE (+)	1.25 (0.98–1.59)	0.074
SVI (+)	1.33 (0.98–1.82)	0.070
Surgical margin (+)	1.89 (1.49–2.39)	<0.001
Four-group categorization		
Upgraded GS 3+4	0.50 (0.35–0.70)	<0.001
Nonupgraded GS 3+4	Reference	<0.001
Upgraded GS 4+3	1.20 (0.86–1.68)	0.274
Nonupgraded GS 4+3	1.55 (1.19–2.02)	0.001

CI: confidence interval, ECE: extracapsular extension; GS: Gleason score; HR: hazard ratio; PSA: prostate-specific antigen; SVI: seminal vesicle invasion.
